# Bruno-3 regulates sarcomere component expression and contributes to muscle phenotypes of myotonic dystrophy type 1

**DOI:** 10.1242/dmm.031849

**Published:** 2018-05-21

**Authors:** Lucie Picchio, Vincent Legagneux, Stephane Deschamps, Yoan Renaud, Sabine Chauveau, Luc Paillard, Krzysztof Jagla

**Affiliations:** 1 GReD (Genetics, Reproduction and Development Laboratory), INSERM 1103, CNRS 6293, University of Clermont Auvergne, 28 Place Henri Dunant, 63000 Clermont-Ferrand, France; 2 IGDR (Institut de Génétique et Développement de Rennes), UMR 6290 CNRS, Université de Rennes, 2 Avenue Léon Bernard, 35000 Rennes, France; 3 Inserm UMR1085 IRSET, Université de Rennes 1, 35000 Rennes, France; 4 CNRS-Université de Rennes1-INRIA, UMR6074 IRISA, 35000 Rennes, France

**Keywords:** Bruno-3, RNA CLIP, CELF1, *Drosophila*, mRNA stability, Myotonic dystrophy type 1

## Abstract

Steinert disease, or myotonic dystrophy type 1 (DM1), is a multisystemic disorder caused by toxic noncoding CUG repeat transcripts, leading to altered levels of two RNA binding factors, MBNL1 and CELF1. The contribution of CELF1 to DM1 phenotypes is controversial. Here, we show that the *Drosophila* CELF1 family member, Bru*-*3, contributes to pathogenic muscle defects observed in a *Drosophila* model of DM1. Bru-3 displays predominantly cytoplasmic expression in muscles and its muscle-specific overexpression causes a range of phenotypes also observed in the fly DM1 model, including affected motility, fiber splitting, reduced myofiber length and altered myoblast fusion. Interestingly, comparative genome-wide transcriptomic analyses revealed that Bru-3 negatively regulates levels of mRNAs encoding a set of sarcomere components, including *Actn* transcripts. Conversely, it acts as a positive regulator of Actn translation. As CELF1 displays predominantly cytoplasmic expression in differentiating C2C12 myotubes and binds to *Actn* mRNA, we hypothesize that it might exert analogous functions in vertebrate muscles. Altogether, we propose that cytoplasmic Bru-3 contributes to DM1 pathogenesis in a *Drosophila* model by regulating sarcomeric transcripts and protein levels.

## Abstract (precis)

**Summary:** Our data suggest that, the so far ignored, cytoplasmic Bru-3/CELF1 contributes to myotonic dystrophy type 1 pathogenesis in a *Drosophila* model by regulating sarcomeric transcripts and protein levels.

## INTRODUCTION

The finding that 75% of human disease-related genes have functional orthologs in the fruit fly (Rubin et al., 2000) drove the development of *Drosophila* models for inherited disorders, including neuromuscular diseases (Chartier et al., 2006; Shcherbata et al., 2007; Garcia-Lopez et al., 2008) such as myotonic dystrophy type 1 (DM1) (de Haro et al., 2006; Yu et al., 2011; Picchio et al., 2013). DM1, which affects 1/8000 people worldwide, is an autosomal dominant disease caused by an unstable expansion of CTG repeats in the 3′ untranslated region (3′UTR) of the *DMPK* gene on chromosome 19 (Brook et al., 1992; Fu et al., 1992). A peculiarity of DM1 is its multisystemic feature – patients display symptoms ranging from baldness and cataract to myotonia, muscle weakness/loss, heart block, sterility, digestive disorders and DM1 type 2 diabetes (Fardaei et al., 2002). Importantly, the severity of symptoms is positively correlated with the size of CTG expansion (Kroksmark et al., 2005), which can vary from 50 to several thousand triplet repeats in the most severe congenital form of DM1. It is well accepted that in muscle cells, mutated *DMPK* transcripts with large CUG expansion form secondary structures (Mooers et al., 2005) able to sequester the muscleblind-like 1 (MBNL1) splicing factor into foci within nuclei (Taneja et al., 1995; Davis et al., 1997). The important role of this factor for DM1 pathogenesis has already been demonstrated in transgenic mouse (Kanadia et al., 2006) and fly (de Haro et al., 2006; Picchio et al., 2013) models. Furthermore, by an as-yet undetermined mechanism, PKCα (PRKCA) is hyperactivated and stabilizes the splicing factor CELF1 (CUGBP, Elav-like family member 1, also known as CUGBP1) through hyperphosphorylation (Kuyumcu-Martinez et al., 2007). MBNL1 and CELF1 play antagonistic roles in regulating the alternative splicing of *CLCN1* (Charlet-B et al., 2002; Kino et al., 2009), *InR* (Kino et al., 2009; Savkur et al., 2004) and *cTNT* (*up*) (Philips et al., 1998; Tran et al., 2011) transcripts. The reverse balance of MBNL1 and CELF1 in DM1 leads to the mis-splicing of these pre-mRNAs, collectively explaining myotonia, diabetes and reduced myocardial function manifested in patients. Some other transcripts, such as *alpha-actinin* (*Actn1-4*) (Suzuki et al., 2002), *MYH14* (Rinaldi et al., 2012) or *Tau* (*MAPT*) (Dhaenens et al., 2011) have been shown to be specifically mis-spliced by CELF1 in DM1. In addition to its role in the regulation of alternative splicing (Philips et al., 1998; Ladd et al., 2001, 2005), CELF1 is also involved in regulating translation (Timchenko et al., 1999; Iakova et al., 2004), mRNA deadenylation and decay (Paillard et al., 2003; Vlasova and Bohjanen, 2008; Lee et al., 2010; Le Tonquèze et al., 2010; Rattenbacher et al., 2010), and RNA editing (Anant et al., 2001). A recent report revealed that CELF1 and MBNL1 antagonize not only to control pre-mRNA splicing, but also to control mRNA stability (Wang et al., 2015). Therefore, CELF1 accumulation in DM1 patients can lead to various alterations in transcript processing.

The role of CELF1 in the DM1-related heart and skeletal muscle disorders has been shown in short-lived mouse models overexpressing Celf1 (Timchenko et al., 2004; Wang et al., 2007). It has also been demonstrated that adult flight muscle degeneration in inducible *Drosophila* DM1 lines can be worsened by overexpressing human CELF1 (de Haro et al., 2006). However, the role of the *Drosophila* CELF1 counterpart and its impact on DM1-associated muscle phenotypes has not yet been investigated. Among the three *Drosophila* genes related to CELF1, i.e. *arrest* or *bruno* (*aret/bru*; *bruno-1*), *bruno-2* (*bru-2*) and *bruno-3* (*bru-3*) (Good et al., 2000; Delaunay et al., 2004), the protein encoded by the ubiquitously expressed *bru-3* is the only one that carries both the RNA recognition motif (RRM) and the linker-specific motif (lsm) (Delaunay et al., 2004), both important for RNA-binding specificities. Bru-3 is also the only Bruno protein capable of binding the EDEN motif, a conserved translational repression element (Delaunay et al., 2004). Thus, we hypothesized that *bru-3* represents a CELF1-like gene in *Drosophila* and tested whether it contributes to DM1 pathogenesis by analyzing the effects of muscle-targeted expression of Bru-3 in fly. We recently generated a set of inducible site-specific *Drosophila* DM1 lines expressing an increasing number of noncoding CUG repeats in larval somatic muscles (Picchio et al., 2013). Among them, the high repeat number *DM1_960_* line that carries 960 interrupted CTG repeats displays particularly severe muscle phenotypes mirroring those observed in DM1 patients. Here, by comparing somatic muscle phenotypes in the DM1_960_ *Drosophila* line, a *bru-3*-overexpressing line and the DM1_960_ line combined with a hemizygous *bru-3* deficiency, we show that the increased level of Bru-3 alters motility and is involved in reduced myofiber length and myoblast fusion. However, we also found that the muscle hypercontraction induced by the expression of the high number of CTG repeats is not Bru-3 dependent. Interestingly, genome-wide transcriptomic analysis performed on larvae with increased muscle levels of Bru-3 identified the downregulation of a large set of genes encoding sarcomere components. Among them, the sarcomeric transcripts encoded by *Actn* were found to be associated with cytoplasmic granules, some of which also colocalize with cytoplasmic Bru-3. As modulating Bru-3 has an opposite effect on *Actn* RNA versus Actn protein levels, we propose that cytoplasmic Bru-3 plays a dual role in DM1. First, increased Bru-3 promotes *Actn* transcript release from the granules and, second, it favors their subsequent *in situ* translation (close to the site of protein incorporation) and a quick post-translational decay (fast mRNA degradation after its translation).

Thus, our data suggest that Bru-3 not only negatively regulates amounts of stored sarcomeric transcripts but also acts as a positive regulator of their *in situ* translation.

## RESULTS

### The *Drosophila* CELF family member, Bru-3, is expressed in larval somatic muscles

**Fig. 1.**
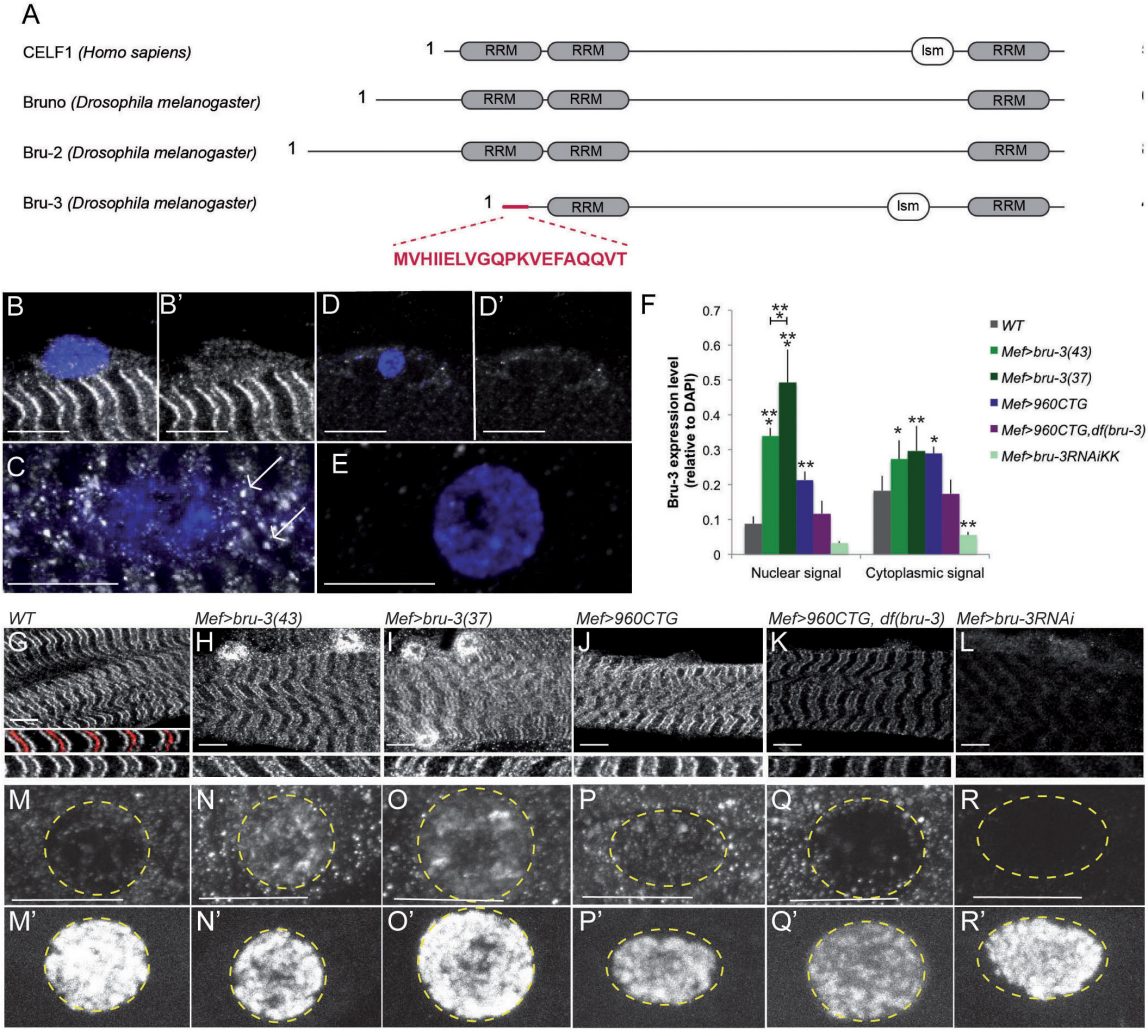
***Bru-3* is expressed in larval somatic muscles and enriched in pathological lines.** (A) CELF1 sequence aligned with Bruno orthologs. lsm, linker-specific motif; RRM, RNA recognition motif. The N-terminus peptide designed to raise the antibody specifically against Bru-3 is highlighted in red. (B-E) Bru-3 expression in WT larval muscle. Immunostaining against Bru-3 (gray) shows that Bru-3 is expressed in larval somatic muscles more specifically in the sarcomeres (B,B′) as well as in granules around the nuclei, as indicated by white arrows (C). This antibody also detected weak Bru-3 expression in nuclei (B′). (D-E) Immunostaining of larval muscles with pre-immune serum shows the specificity of the antibody raised against Bru-3. (F) Densitometric measurements of Bru-3 in the nucleus and cytoplasm of different genotypes. Cytoplasmic signal measurement was performed using ImageJ as described previously by McCloy et al. (2014). DAPI was used as reporter of tissue accessibility for the staining, because sarcomeric markers expression is potentially altered in pathological contexts (see Fig. 4). *P<0.05, **P<0.01, ***P<0.001 versus WT. (G-L) *Bru-3* expression in segment border muscle (SBM) of third-instar larvae. Sarcomeric immunostained Bru-3 (gray) frames the Z-line represented by Actn immunostaining (red) in WT muscle (G). Bru-3 immunostaining is also shown in *Mef>bru-3(43)* (H), *Mef>bru-3(37)* (I), *Mef>960CTG* (J), *Mef>960CTG,Df(bru-3)* (K) and *Mef>bru-3RNAi* (L) conditions. (M-R) Representative pictures of nuclear Bru-3 expression compared with corresponding DAPI staining (M′-R′) in third-instar larval muscle. Bru-3 immunostaining is shown in WT (M), *Mef>bru-3(43)* (N), *Mef>bru-3(37)* (O), *Mef>960CTG* (P), *Mef>960CTG,Df(bru-3)* (Q) and *Mef>bru-3RNAi* (R). Scale bars: 20 µM.

Alignment of protein domains of human and *Drosophila* CELF family members (Fig. 1A) revealed that Bru-3 conserves both RRM and lsm domains (Delaunay et al., 2004). Alignment of the lsm domain of human CELF proteins and *Drosophila* Bruno proteins (Fig. S1) showed that only CELF1, CELF2 and Bru-3 lsm domains are well conserved (95-100%) compared with other CELF and Bruno proteins, for which conservation ranges from 62% to 14%. In addition, Aret/Bru which is known to be expressed in a subset of adult muscles (Oas et al., 2014; Spletter et al., 2015) is not expressed in *Drosophila* larval muscles as shown by immunostaining (Fig. S1B,C), making it unlikely that it is a functional CELF1 ortholog. Thus, among the Bru genes, *bru-3* appears to be the closest *Drosophila* CELF1 homolog. To characterize the *bru-3* expression pattern, we generated an antibody raised against the Bru-3 N-terminus end (Fig. 1A), a very specific portion of this protein. We found that Bru-3 protein is detected in larval body wall muscles and displays a cytoplasmic striated pattern (Fig. 1B,G) that frames the Z-line revealed by Actinin (Actn) immunostaining in red (Fig. 1G). Interestingly, other RNA-binding proteins, including the involved-in-DM1 splicing factor Muscleblind (Mbl) (Llamusi et al., 2013) and fragile X-related protein 1 (FXR1) (Khandjian et al., 1998; Mientjes et al., 2004; Whitman et al., 2011), are expressed in sarcomeres. A higher-magnification view (Fig. 1C) shows that cytoplasmic Bru-3 is also found in granules around nuclei. These granules can be observed around all nuclei. Finally, low levels of Bru-3 are found in the nuclei of muscle fibers (Fig. 1B,B′), reflecting its potential role as a splicing factor. These immunostaining data are also supported by western blot analysis results (Fig. S1E), revealing Bru-3 protein in both cytoplasmic and nuclear fractions. That the generated antibody specifically detects Bru-3 protein is supported by the lack of pre-immune serum staining (Fig. 1D-E), the reduced intensity of Bru-3 fluorescent signal in muscles dissected from *bru-3 RNAi* knockdown larvae (*Mef>bru-3RNAi*) (Fig. 1F,L,R) and by western blot detection of Bru-3 protein (Fig. S1E). Muscle-targeted overexpression of *bru-3* leads to increased signal intensity in the cytoplasm (Fig. 1F,H,I; Fig. S1E) combined with a higher nuclear accumulation of Bru-3 (Fig. 1F,N,O). Among two *bru-3*-overexpressing lines, quantification of immunostaining revealed that *UAS-bru-3(37)* has a significantly higher nuclear level of Bru-3 than has *UAS*-*bru-3(43)* (Fig. 1F). When considering *bru-3* transcript expression, in *Mef>bru-3(37)* context, they were highly upregulated but remained at control levels in *DM1_960_* pathogenic context (Fig. S1D).

As CELF1 is increased in DM1 cardiac and skeletal muscles of both patients and mice (Savkur et al., 2001; Wang et al., 2007; Orengo et al., 2008), we assessed Bru-3 expression in larval muscles of pathological *Drosophila* lines. We observed that Bru-3 expression pattern in muscles of DM1_960_ larvae [a high-repeat-number line that carries 960 interrupted CTG repeats (Picchio et al., 2013)] is similar to the wild-type (WT) pattern (Fig. 1G,J). However, we recorded a significantly increased intensity of the fluorescent signal detected by anti-Bru-3 antibody in the cytoplasm (Fig. 1F,J) and the nuclei (Fig. 1F,P). We also found that Bru-3 signal intensity is rescued in DM1_960_ muscles with hemizygous *bru-3* deficiency (Fig. 1F,K,Q; Fig. S1E, Table S1). Hence, the DM1_960_ line not only mimics the muscle phenotype of DM1 patients (Picchio et al., 2013) but also increased the protein levels of its CELF1 counterpart. Thus, our DM1 and *bru-3*-overexpressing lines appear suitable for comparative studies and assessing *CELF1/bru-3*-dependent pathological phenotypes in DM1.

### Increased Bru-3 levels contribute to impaired motility and muscle morphology defects in DM1, but not to muscle hypercontraction

**Fig. 2.**
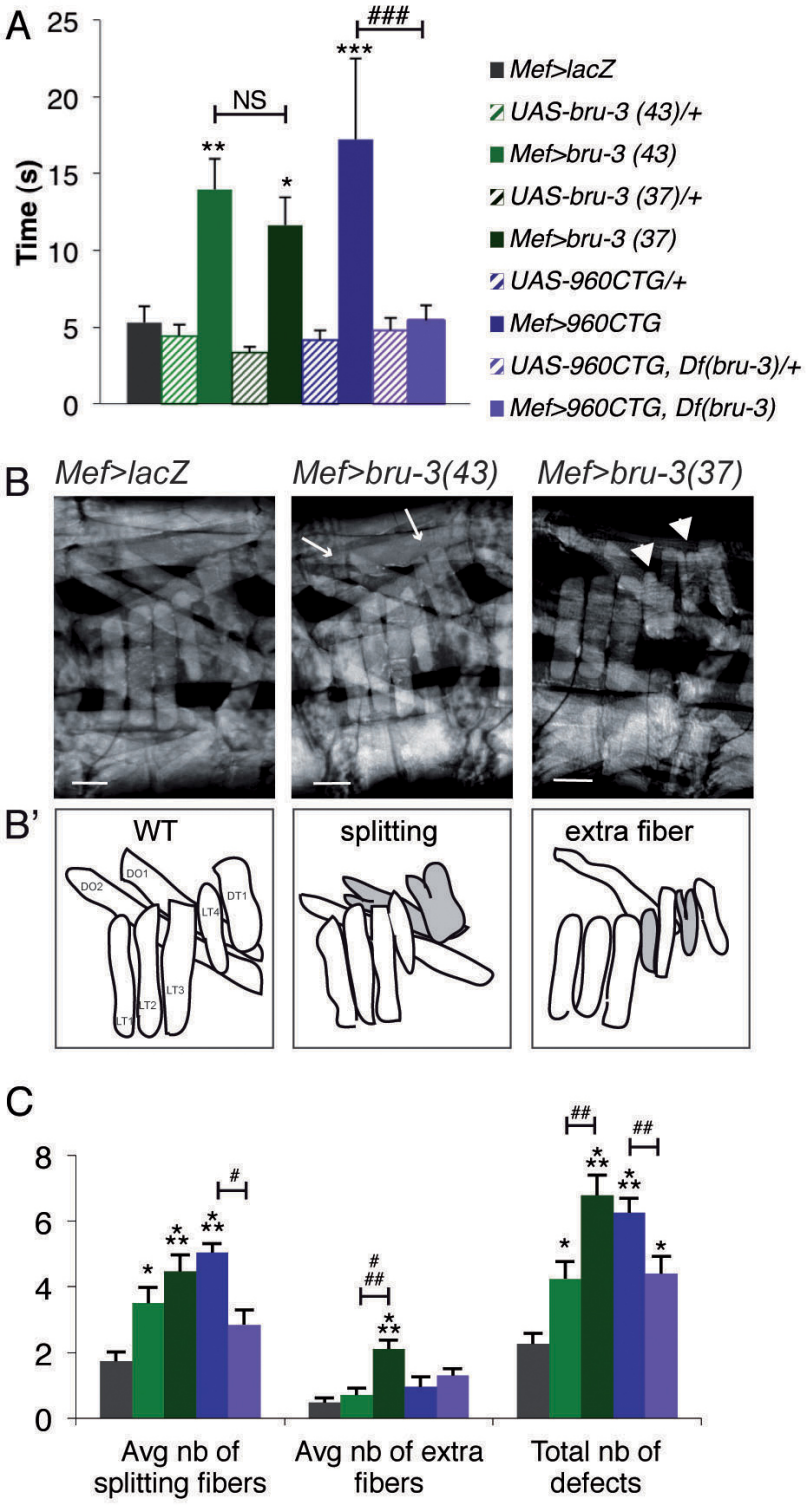
***Bru-3* overexpression alters motility and contributes to fiber splitting.** (A) Righting assay. The graph represents the average recorded time taken for the larvae of each genotype to turn over. (B-C) Assessment of overall muscle pattern and quantification of muscle abnormalities. Z-stacks of *in vivo* scans of muscle pattern in a single third-instar larvae abdominal segment (between A5 and A7) (B). Arrows point to splitting fibers and arrowheads to extra fibers, both represented by schemes in B′. Muscles taken into account for quantification are represented and named in WT context (B′): LT1, LT2, LT3 and LT4 refer to lateral transverse muscles; DT1, dorso transverse 1; DO1 and DO2, dorso oblique muscles. Graphs show the average number of each defect observed *in vivo* for each mutant line on a window of three abdominal segments (C). Color coding is as in A. *P<0.05, **P<0.01, ***P<0.001 versus *Mef>lacZ*. NS, not significant. ^#^*P*<0.05, ^##^*P*<0.01, ^###^*P*<0.001 *Mef>960CTG* versus *Mef>960CTG,Df(bru-3)* or *Mef>bru-3(37)* versus *Mef>bru-3(43)*. Scale bars: 70 µM.

To assess the functionality of muscles with increased Bru-3 levels, we performed a righting assay by putting a larva on its back and recording the time it takes to revert to ventral position. We observed that all control lines needed ∼5 s to complete this exercise, whereas *Mef>bru-3(37)*, *Mef>bru-3(43)* and *DM1_960_* lines needed 12, 14 and 17 s, respectively (Fig. 2A). The hemizygous *bru-3* deficiency, which reduces Bru-3 levels (Fig. 1K), completely restores the motility of the *DM1_960_* line (Fig. 2A). To test whether muscle defects are at the origin of impaired larva motility, we scored morphological abnormalities in the body-wall musculature of third-instar larvae. The main defects identified were splitting fibers and extra fibers (Fig. 2B,B′), with extra fibers already defined as a consequence of extreme muscle fiber splitting (Picchio et al., 2013). The total number of defects observed (including splitting, extra fibers and missing fibers) was significantly increased in the pathological lines compared with the *Mef>lacZ* control line (Fig. 2C). Note that the number of defects was higher in the strongest *bru-3*-overexpressing line, *Mef>bru-3(37)*, than in the *Mef>bru-3(43)* line, despite an absence of difference of righting between those two lines. Thus, the former line was used for subsequent experiments. We also noted a significantly reduced number of splitting fibers and overall defects in the *DM1_960_,Df(bru-3)* rescue line compared with the *DM1_960_* line (Fig. 2C), suggesting that Bru-3 accumulation in DM1 induces the splitting fiber phenotype. Taken together, these data indicate an important contribution of Bru-3 to the altered muscle performance and morphological muscle defects observed in our *Drosophila* DM1 model.

**Fig. 3.**
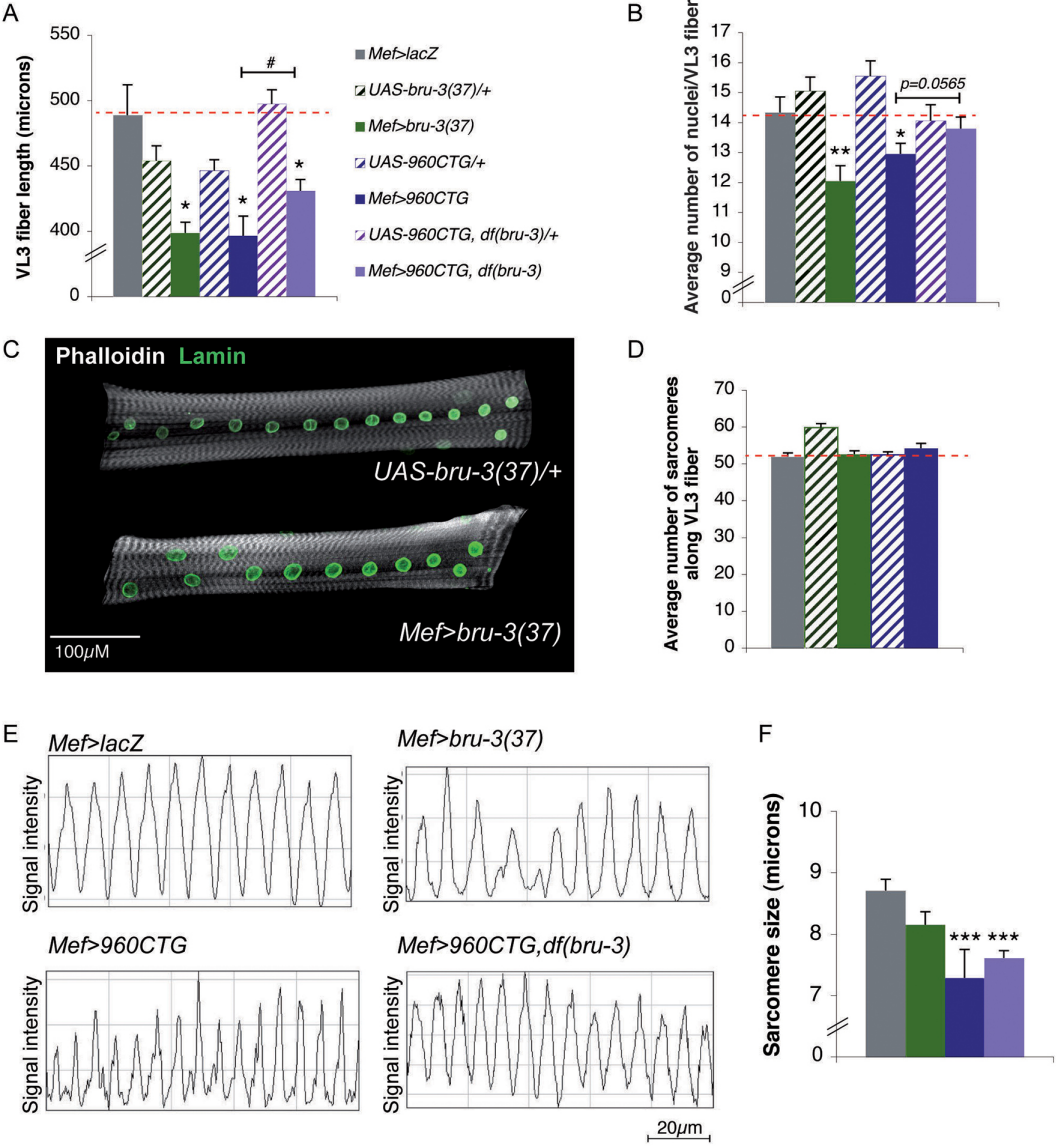
***bru-3* overexpression affects myoblast fusion process, but not contractility, *in vivo*.** (A) The average length of abdominal VL3 fibers is significantly reduced in *Mef>bru-3(37)* and *Mef>bru-3(43)* lines, and in *Mef>960CTG* and *Mef>960CTG,Df(bru-3)* lines, compared with controls (*Mef>lacZ* and corresponding transgenic control line). (B,C) Myoblast fusion defect in DM1 condition is Bru-3 dependent. The average number of nuclei per abdominal VL3 fiber is significantly reduced in *Mef>bru-3* and *Mef>960CTG* lines, but is rescued in *Mef>960CTG,Df(bru-3)* condition (B). Number of nuclei is used as an indicator of the number of fusion events during myogenesis. Fusion is therefore affected in the DM1 line in a Bru-3-dependent manner. Images of abdominal VL3 fibers representative of an altered condition [*Mef>bru-3(37)*] and control condition [*UAS-bru-3(37)/+*] (C). Nuclei were stained with anti-Lamin antibody (green). Actin was stained with phalloidin (gray). (D) Larval muscle growth was not affected in pathological contexts. The average number of sarcomeres along the VL3 fiber is represented for each genotype and represents an index of fiber growth. (E,F) Sarcomere shortening in DM1 condition is not Bru-3 dependent. Z-band profiles along VL3 fibers were assessed with phalloidin staining along a 100-µM length (E). The distance measured between two peaks gives sarcomere size. Thus, more peaks present on the profiles equates to more contracted muscle. *P<0.05, **P<0.01 versus both *Mef>lacZ* and respective transgenic control line. Sarcomere size, which reflects the state of contraction or relaxation of VL3 muscle, is presented on the graph for each mutant line (F). A significant reduction in the length of a sarcomere is an index of muscle hypercontraction. ***P<0.001.

It is well known that in cell culture, the fusion ability of DM1 satellites cells (Furling et al., 2001; Thornell et al., 2009) and expanded CUG repeat tract-expressing C2C12 myoblasts is altered (Furling et al., 2001; Amack and Mahadevan, 2001) and impacts on myotube size. As previously observed (Picchio et al., 2013), the larval ventrolateral (VL3) fibers were shorter and displayed a significantly reduced number of nuclei in DM1 lines (*Mef>960CTG*, Fig. 3A,B). The same was true in the *Mef>bru-3(37)* line (Fig. 3A,B). Importantly, fiber length and number of nuclei were partially restored in *DM1_960_,Df(bru-3)* larvae (Fig. 3A,B), indicating that *bru-3* overexpression contributes to muscle fusion phenotypes observed in DM1 lines. One can note that nuclei are normally distributed along the fiber in *bru-3* overexpression context (Fig. 3C). Fig. 3D shows that the number of sarcomeres along muscle fibers remains unchanged in pathological lines compared with controls, meaning that fiber growth defects cannot account for reduced fiber size, as can reduced number of nuclei. Thus, both the expression of CUG repeats and *bru-3* overexpression affect muscle fibers length resulting from reduced number of myoblast fusion events.

As previously described (Picchio et al., 2013), systematic quantification of sarcomere size following Z-line staining with phalloidin in several larvae revealed muscle hypercontraction in the *Mef>960CTG* line (Fig. 3E,F). In contrast, the sarcomere size was not significantly affected in the *bru-3* overexpression line compared with the *Mef>lacZ* control line (Fig. 3E,F). This result suggests that *bru-3* overexpression does not affect muscle relaxation. In line with this observation, sarcomere size is still reduced in the *DM1_960_,Df(bru-3)* line, as it is in the *DM1_960_* line, which indicates that Bru-3 is not involved in the hypercontracted muscle phenotype in the *Drosophila* model of DM1, much like its vertebrate counterpart, CELF1 (Ward et al., 2010).

### mRNA profiling of *bru-3*-overexpressing and DM1 lines reveals *bru-3*-dependent deregulation of genes encoding sarcomeric components

**Fig. 4.**
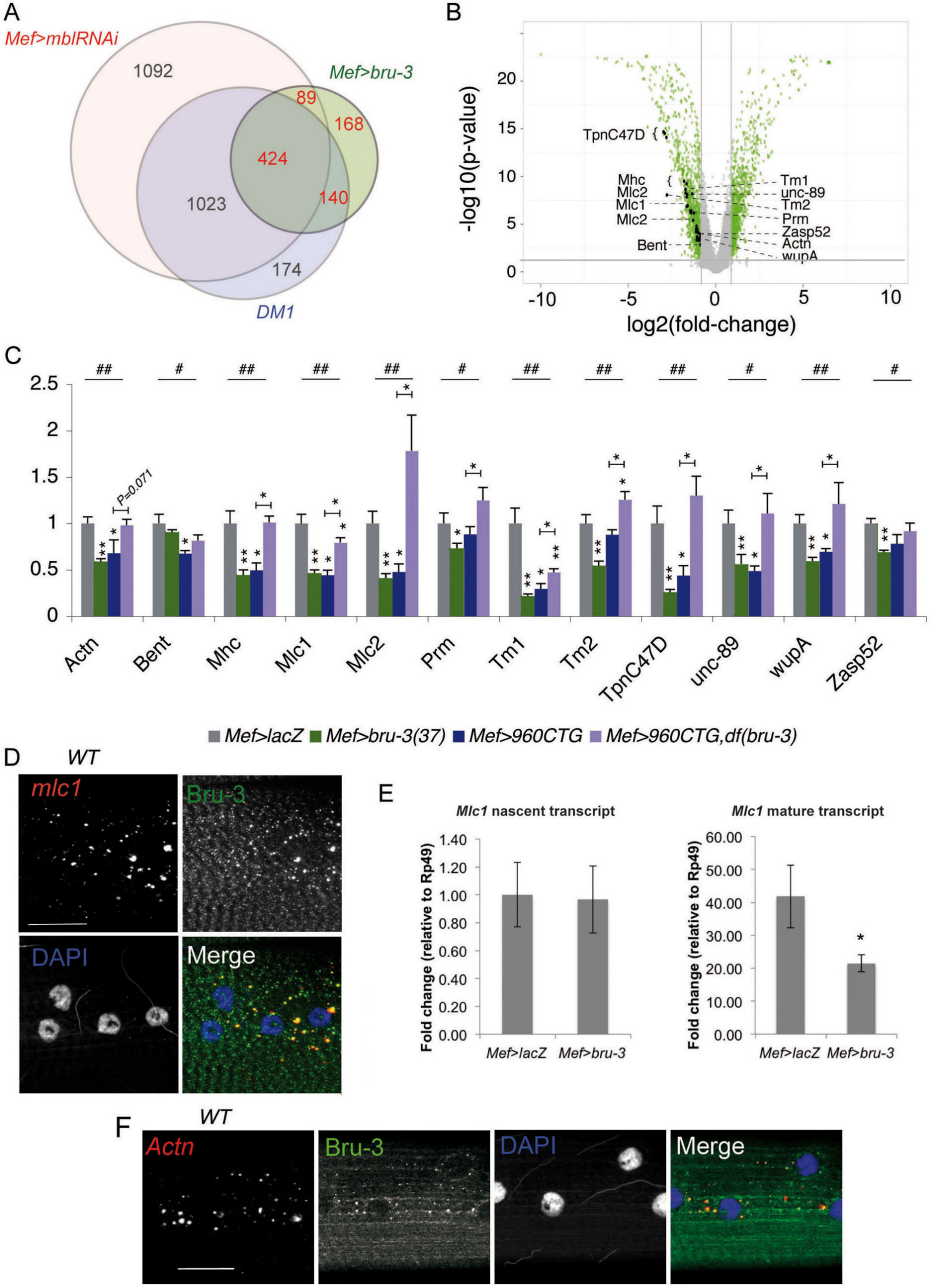
**Global gene expression analysis of *bru-3* overexpression suggests a cytoplasmic function for Bru-3 in regulating sarcomeric transcript stability.** (A) Venn diagrams of genes downregulated in *Mef>bru-3(37)* condition versus *Mef>mblRNAi* and DM1 (*Mef>960CTG*∩*Mef>600CTG*) lines show that ∼80% of transcriptomic alterations caused by Bru-3 overexpression are common to DM1 and/or *mbl*-attenuated lines. The diagram was generated from a list of transcripts that are >1.5-fold enriched or depleted relative to the *Mef>lacZ* reference. (B) Volcano plot summarizing microarray data for the *Mef>bru-3(37)* line versus *Mef>lacZ*. The horizontal axis plots the fold change on a log2 scale. The vertical axis plots the *P*-value on a −log10 scale. Gray dots indicate probes below the threshold. Green dots indicate probes with significantly altered expression. Black dots indicate downregulated sarcomere component probes. (C) Expression of transcripts encoding sarcomeric proteins is Bru-3 dependent. RT-qPCR on a set of mRNAs encoding sarcomeric proteins in normal condition (*Mef>lacZ*), pathological contexts [*Mef>960CTG*, *Mef>bru-3(37)*] and rescue condition [*Mef>960CTG,Df(bru-3)*]. *P<0.05, **P<0.01 versus *Mef>lacZ* or versus *Mef>960CTG* where indicated by a colored bar. ^#^*P*<0.05, ^##^*P*<0.01 indicate significant differences in data distribution between genotypes (Kruskal–Wallis test). (D) Sarcomeric transcript *Mlc1* (red) colocalizes with Bru-3 (green) in cytoplasmic granules surrounding the DAPI-stained nuclei (blue) in WT condition. Scale bar: 20 µM. (E) qPCR of nascent *Mlc-1* and mature *Mlc1* transcripts indicates that despite a reduced level of mature transcripts, transcription is at the same rate between control (*Mef>lacZ*) and *bru-3*-overexpressing lines. *P<0.05 versus *Mef>lacZ*. (F) Sarcomeric *Actn* transcripts (red), similar to *Mlc1* transcripts (D), colocalize with Bru-3 (green) in cytoplasmic granules. DAPI-stained nuclei are in blue. Scale bar: 60 µm.

The above data show that *bru-3* overexpression contributes to impaired motility and muscular defects in the DM1 line. We carried out a transcriptomic analysis to obtain a more global picture of how far Bru-3 contributes to CTG repeat-induced gene deregulations. By profiling gene expression in the strongest *bru-3*-overexpressing line, *Mef>bru-3(37)*, we identified 396 upregulated and 451 downregulated genes (Table S2). We compared this repertoire of genes with the microarray data from our previous work [*Mef>mblRNAi*, *Mef>600CTG* and *Mef>960CTG* (Picchio et al., 2013)]. Comparison of the data sets was facilitated by the fact that we used the same microarray platform and the same controls. As shown in the Venn diagrams in Fig. 4A, 32% of the genes downregulated in DM1 are common to the *bru-3* overexpression condition, compared with 82% for *mbl* attenuation. For the upregulated genes, *Mef>bru-3* represents 53% of the deregulations against 70% for *Mef>mblRNAi* (Fig. S2A). Hence, the extent of gene deregulation indicates that the *bru-3*-dependent transcriptomic alterations in DM1 lines are less pronounced than *mbl*-dependent alterations. Interestingly, 8% of the genes downregulated, and 9% of the genes upregulated, in the DM1 line are dependent on *bru-3* overexpression but not on *mbl* attenuation (Fig. 4A; Fig. S2A). Thus, the global gene deregulation in *bru-3* overexpression illustrates its partial contribution to DM1 phenotypes.

A gene ontology (GO) classification of the genes downregulated (Fig. S2B) and upregulated (Fig. S2C) in muscle-targeted *bru-3* overexpression showed that genes implicated in redox processes and genes encoding sarcomeric proteins were mainly downregulated. As deregulation of sarcomere components might weaken muscle structure and lead to splitting of muscle fibers, a phenotype observed in both DM1 patients and our DM1 *Drosophila* model, we focused on this category of genes (Fig. S2B, Table 1). As charted on the volcano plot (Fig. 4B), we found that the increased level of Bru-3 leads to downregulation of 11 genes encoding sarcomeric proteins including *α-Actinin* (*Actn*), *Myosin heavy* and *light chains* (*Mhc*, *Mlc1* and *Mlc2*), *Tropomyosin 1* and *2* (*Tm1* and *Tm2*), *Troponin I* (*wupA*) and *C47D* (*TpnC47D*), *bent*, *Paramyosin* (*Prm*), *Zasp52* and *Unc-89.*

**Table 1.**
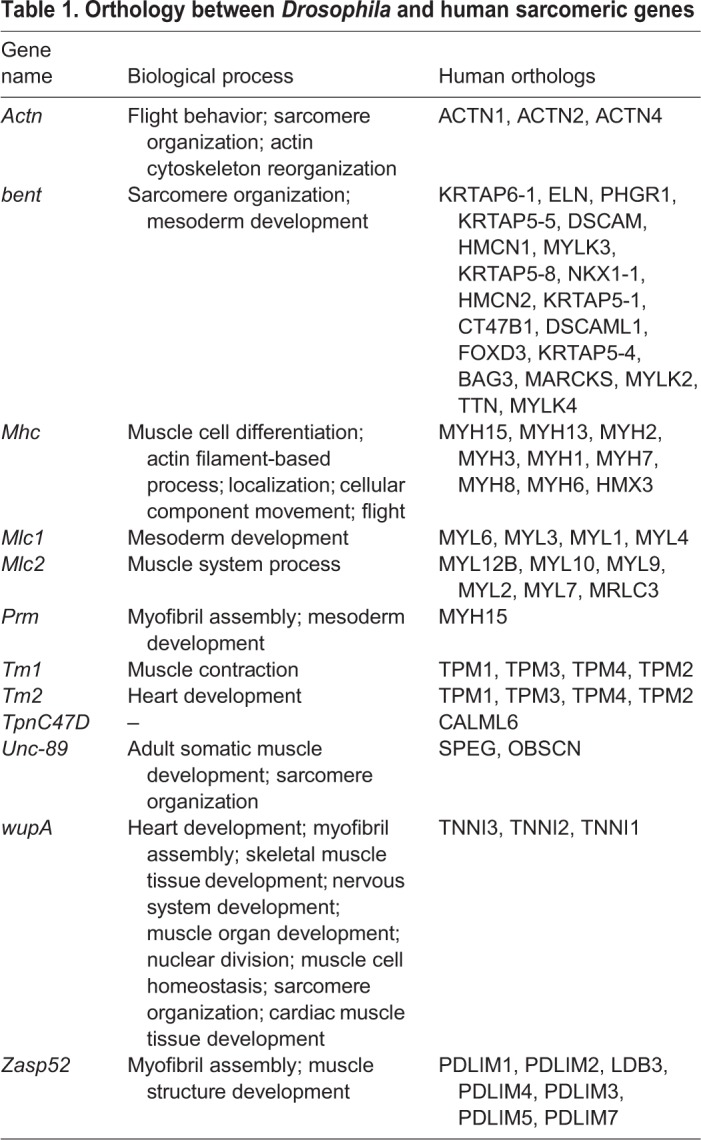
**Orthology between *Drosophila* and human sarcomeric genes**

This finding raised the possibility that Bru-3 is directly involved in the observed DM1-induced repression of sarcomeric genes. To investigate this possibility, we first used reverse transcription (RT)- quantitative polymerase chain reaction (qPCR) to test whether the identified sarcomeric transcripts are indeed downregulated. In *bru-3* overexpression, all candidates except *bent* showed significantly decreased expression (Fig. 4C), thus validating the global gene expression data. We also confirmed that expression of *Actn*, *Mhc*, *Mlc1*, *Mlc2*, *Tm1*, *TpnC47D*, *Unc-89* and *wupA* (eight of the 11 transcripts downregulated in microarrays) was significantly decreased in DM1. Finally, we demonstrated that decreasing Bru-3 in DM1 context by combining the DM1 line with *bru-3* deficiency partially or totally rescued sarcomeric transcript levels, providing evidence for *bru-3*-dependent downregulation of sarcomeric genes in the *Drosophila* DM1 model. Interestingly, crosslinking immunoprecipitation (CLIP) followed by sequencing (CLIP-seq) experiments performed on mouse muscle tissues by Wang et al. (2015) also identified sarcomeric genes as direct targets of CELF1. Among them, orthologs of *bent* (*Eln*, *Bag3*, *Mylk2*, *Ttn*), *Mhc* (*Myh1*), *Mlc1* (*Myl1*, *Myl3*), *Tm1* and *Tm2* (*Tpm2*), *Unc-89* (*Speg*) and *Zasp52* (*Ldb3*, *Pdlim3*, *Pdlim5*, *Pdlim7*) sorted out.

The observed lower levels of sarcomeric mRNAs in *bru-3* overexpression suggested that *bru-3* overexpression could affect sarcomeric mRNAs transcription or stability. We favor the idea that Bru-3 controls sarcomeric mRNA stability for several reasons. First, mammalian CELF1 has been shown to bind 3′UTRs of target transcripts and regulate their stability (Paillard et al., 2003; Vlasova and Bohjanen, 2008; Wang et al., 2015; Masuda et al., 2012), and this function might be conserved in *Drosophila*. Second, fluorescent *in situ* hybridization (FISH) of *Mlc1* followed by Bru-3 immunostaining (Fig. 4D) showed that *Mlc1* mRNA colocalizes with Bru-3 in WT larval muscles in granular cytoplasmic structures, consistent with an interaction between *Mlc1* mRNA and Bru-3. Last, but not least, we found that the nascent *Mlc1* transcripts quantified using intron-specific RT-qPCR are present at the same level in *bru-3*-overexpressing and WT larval muscles, despite a reduced level of mature *Mlc1* RNA in the first condition (Fig. 4E). Hence, Bru-3 does not interfere with *Mlc1* transcription. Taken together, these data suggest that Bru-3 controls the abundance of *Mlc1* and potentially other sarcomeric transcripts by a post-transcriptional mechanism.

We next explored the nature of granular structures in which *Mlc1* RNA and Bru-3 colocalize. As CELF1 has been shown to recruit occludin mRNA or E-cadherin mRNA to P-bodies (Yu et al., 2013, 2016), we hypothesized that they correspond to P-bodies. Indeed, Bru-3 partially colocalizes with Me31B and FMRP, two P-body markers (Fig. S2D,E). As P-bodies are associated with mRNA storage, distribution and decay, we suggest that Bru-3 controls sarcomeric mRNA stability in a P-body-dependent manner.

### Conservation of CELF1 interaction with sarcomeric transcripts in C2C12 myotubes

**Fig. 5.**
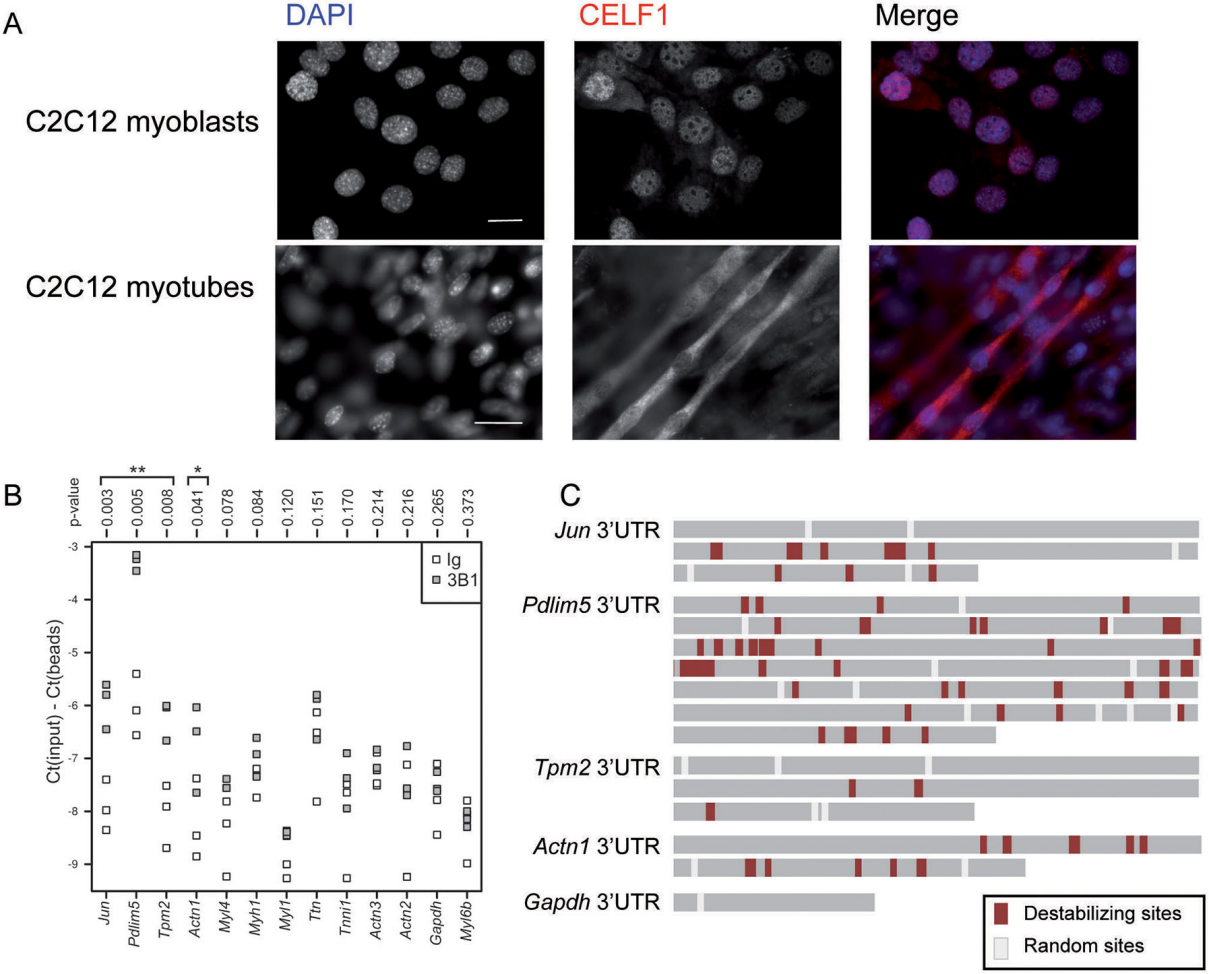
**The Bru-3 ortholog, CELF1, is relocalized in the cytoplasm of C2C12 myotubes and binds mRNA transcripts orthologous to Bru-3 targets.** (A) Immunostaining against CELF1 (using 3B1 antibody) in undifferentiated C2C12 myoblasts and C2C12 myotubes after 10 days of differentiation. CELF1 signal (red) is essentially nuclear in myoblasts and accumulates in the cytoplasm upon differentiation. Nuclei are DAPI counterstained (blue). Scale bars: 20 µM. (B) Sarcomeric transcripts physically bound to CELF1 in myotubes. Strip charts show the enrichments of cross-linked mRNAs in immunopurified complexes (CLIP experiments). Enrichments on anti-CELF1 beads (gray boxes) are compared with those on IgG beads (white boxes). Student’s *t*-test *P*-values are shown above each lane of the graph. *Jun* mRNA (a known CELF1 target) was used as a positive control; *Gapdh* was used as a negative control. (C) 3′UTR of significantly-enriched sarcomeric transcripts in CLIP experiments. Destabilizing sites (Lee et al., 2010) (Table S3) are represented in red and random sites in white. *Jun* was used as a positive control; *Gapdh* was used as a negative control. *P<0.05, **P<0.01.

To assess whether the observed cytoplasmic expression and function of Bru-3 is conserved in vertebrate muscle cells, we tested CELF1 expression in C2C12 myoblasts and differentiated myotubes. In C2C12 myoblasts, CELF1 is mainly present in nuclei (Fig. 5A). However, after 10 days of differentiation, CELF1 is essentially excluded from the nuclei and relocalized, predominantly in the cytoplasm of myotubes (Fig. 5A). This suggests that, like in *Drosophila*, it exerts mainly cytoplasmic functions in differentiated muscle cells. To test this hypothesis, we performed an RNA CLIP experiment on C2C12 myotubes using anti-CELF1 antibody and RT-qPCR to individually test orthologs (Table 1) of 12 sarcomeric candidates sorted with the *Drosophila* model. Among them, we found that *Pdlim5* (*Zasp52*), *Tpm2* (*Tm1* and *Tm2*) and *Actn1* (*Actn*) transcripts are significantly enriched in CLIP complexes (Fig. 5B; Fig. S3) suggesting that these mRNAs physically interact with CELF1. Interestingly, *in silico* exploration revealed an enrichment of CELF1-dependent destabilizing sites (Lee et al., 2010) in the 3′UTR of these transcripts, as observed in the positive control used for the CLIP (*Jun*; *Jra*) and contrary to the 3′UTR of the negative control (*Gapdh*) (Fig. 5C; Table S3). Consequently, we argue a cytoplasmic role for Bru-3/CELF1 in the post-transcriptional regulation of sarcomeric transcripts in both flies and mammals.

### Actn, an example of Bru-3-dependent regulation of sarcomere components in normal and pathological conditions

**Fig. 6.**
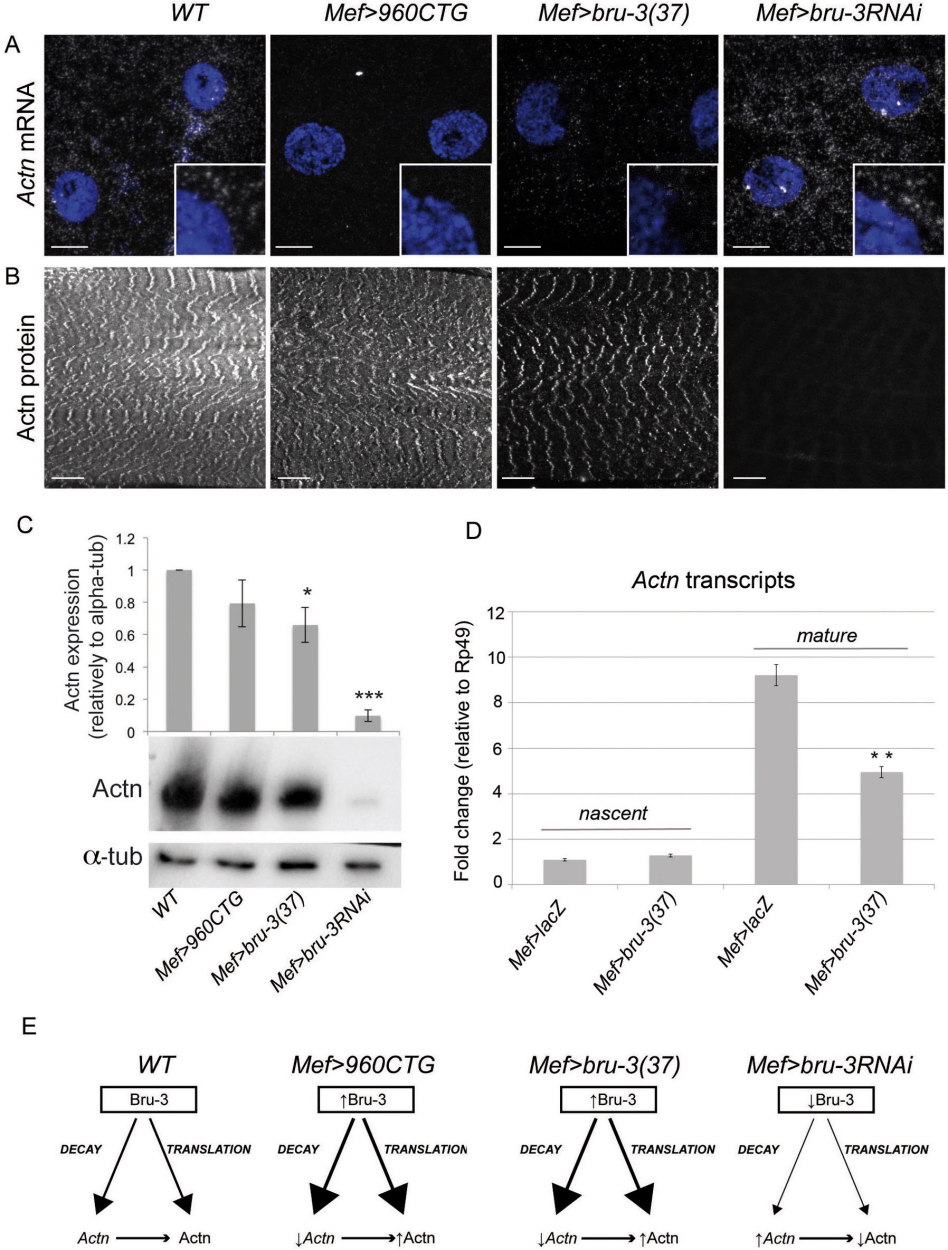
**Modulating Bru-3 level influences Actn transcript and protein levels in an opposite manner.** (A) Single-molecule FISH showing *Actn* transcripts in muscle cytoplasm (gray). Nuclei are visualized with DAPI (blue). Very low and lower *Actn* signals are detected in DM1_960_ and *bru-3*-overexpressing lines, respectively, compared with WT. *Actn* mRNA is enriched in the cytoplasm of the knockdown line muscles. Scale bars: 10 µm. (B) Actn protein immunostaining in the different conditions. Scale bars: 20 µm. (C) Western blot analysis of Actn protein and protein quantification relative to alpha-tubulin (α-tub) immunoblot (*n*=3-4). (D) qPCR of nascent and mature *Actn* transcripts shows that the *Actn* transcription rate remains unchanged between control (*Mef>lacZ*) and *bru-3*-overexpressing lines. **P<0.01 versus *Mef>lacZ*. (E) Models of Bru-3 actions on *Actn* transcripts and on Actn protein levels in normal and pathological conditions.

To further analyze the consequences of sarcomeric transcript deregulation and establish a link between Bru-3 function and DM1, we focused on *Actn*, a conserved sarcomeric target of Bru-3/CELF1. Similar to *Mlc1* mRNA, *Actn* mRNA revealed by a standard *in situ* hybridization protocol was detected in granular cytoplasmic structures scattered around the nuclei and, in part, co-expressing Bru-3 (Fig. 4F). Using a Stellaris single-molecule hybridization protocol to more accurately detect *Actn* transcripts in muscle cytoplasm, we observed a widespread cloud of mRNAs around the nuclei in the WT condition (Fig. 6A). In DM1_960_ and *bru-3*-overexpressing lines, the number of spots detected is decreased owing to reduced *Actn* transcript levels, as demonstrated by microarray (Fig. 4B) and RT-qPCR (Fig. 4C) analyses. On the contrary, in a *bru-3* knockdown context (*Mef>bru-3RNAi*), the *Actn* mRNA signal is homogenously increased in the cytoplasm (Fig. 6A). Thus, these observations suggest that cytoplasmic Bru-3 directly or indirectly promotes the degradation of *Actn* mRNA. Whether nuclear Bru-3 contributes to the negative *Actn* transcript regulation in *Mef>bru-3* context remains to be tested. However, considering that Bru-3 does not regulate *Mlc1* at a transcriptional level (Fig. 4E), and that both *Mlc1* and *Actn* transcripts display similar sensitivity to Bru-3 (Fig. 4C), we hypothesize that *Actn* regulation follows the *Mlc1* scheme and is essentially post-transcriptional.

We next investigated Actn expression in pathological lines at the protein level. Actn displayed a striated pattern in control and pathological lines (Fig. 6B). Surprisingly, as shown by western blotting, changes in Actn protein levels did not follow those of *Actn* transcripts. In Bru-3-accumulating *Mef>960CTG* and *Mef>bru-3* lines characterized by a highly reduced level of *Actn* RNA, reduction in Actn protein levels, even if detectable, was much less pronounced (Fig. 6C). In an opposite way, in the *Mef>bru-3RNAi* line, despite a high *Actn* transcript content in the cytoplasm, the Actn protein in the sarcomeres was dramatically reduced (Fig. 6B,C). Also, only the mature, but not nascent, *Actn* transcripts are reduced in *Mef>bru-3* context (Fig. 6D), supporting post-transcriptional regulation of *Actn* by Bru-3. Altogether, these observations suggest that, in addition to negatively regulating *Actn* transcript storage, Bru-3 also positively regulates Actn protein synthesis. As the polyribosomes have been previously observed associated with sarcomeric myosin thick filaments (Heywood et al., 1967; Allen and Terrence, 1968), we hypothesize that sarcomeric proteins might be translated *in situ* (close to the site of protein incorporation), thus supporting the translation/co-translational assembly model of Zarnescu and Gregorio (2013). Cytoplasmic Bru-3 in the sarcomeres could be involved in positively regulating *in situ* translation (Fig. 6E).

## DISCUSSION

The underlying molecular mechanisms of DM1 are particularly complex. Among them, the disruption of the balance between the splicing factors MBNL1 and CELF1 (Ho et al., 2005; Kuyumcu-Martinez et al., 2007), transcription factor deregulation (Ebralidze et al., 2004; Yadava et al., 2006; Dansithong et al., 2011), or altered maturation of miRNA (Perbellini et al., 2011; Rau et al., 2011) all lead to perturbations in transcript levels. MBNL1 sequestration and CELF1 accumulation are the first and best-studied mechanisms of DM1 pathogenesis, but the specific contribution of CELF1 to DM1-associated phenotypes has not yet been entirely elucidated. As 77% of human genes involved in diseases have an ortholog in *Drosophila melanogaster* (Reiter et al., 2001), we hypothesized that obtaining insights into CELF1 counterpart function in the fly could contribute to elucidating its significance in DM1.

### The *Drosophila CELF1*-like gene, *bru-3*, is expressed in larval muscle and contributes to DM1-associated muscle phenotypes

Studies on mice have been conducted to dissect the involvement of MBNL1 and CELF1 in DM1 (Timchenko et al., 2004; Ho et al., 2005; Kanadia et al., 2006; Wang et al., 2007; Koshelev et al., 2010; Ward et al., 2010; Suenaga et al., 2012). However, understanding the respective contributions of these factors to DM1 phenotypes needs further investigation. Here, we applied a *Drosophila* model to better characterize the functions of CELF1/Bru-3.

Contrary to *mbl* attenuation, which reproduces all muscle phenotypes observed in DM1 larvae (Picchio et al., 2013), *bru-3* overexpression phenocopies only some of them. Furthermore, we observed that reducing Bru-3 in the DM1_960_ line [*DM1_960_,Df(bru-3)*] preserves muscle function but fails to rescue myotonia-related muscle hypercontraction. Interestingly, a similar observation was made in the 5-313^+/^^−^ mouse model of DM1, in which loss of CELF1 preserves muscle function but does not improve myotonia (Kim et al., 2014), thus suggesting that *Drosophila* is a reliable model for studying CELF1 involvement in DM1.

The partial contribution of Bru-3 to DM1 phenotypes is also indicated by the genome-wide analyses revealing that only 32% of the genes downregulated in DM1 contexts are also downregulated after muscle-targeted overexpression of *bru-3*. Among the downregulated genes common to DM1/overexpressed Bru-3, we found those encoding major sarcomeric components, such as Actn or Tpm, which suggests that their depletion could contribute to reduced muscle performance in DM1. As we observed that reducing Bru-3 in DM1 larvae corrected expression levels for most of the downregulated sarcomeric genes, we assume that sarcomeric gene deregulation in the *Drosophila* DM1 model is Bru-3 dependent.

Hence, our study is consistent with previous findings (Wang et al., 2007; Kim et al., 2014; de Haro et al., 2013) showing that *CELF1* overexpression alone does not reproduce all the DM1 phenotypes.

### Bru-3 and CELF1 regulate levels of sarcomere component transcripts

So far, it has been demonstrated that the alterations of MBNL1 or CELF1 in DM1 lead to mis-splicing of some sarcomere component transcripts, e.g. *PDLIM3* (Lin et al., 2006), *MYH14* (Rinaldi et al., 2012), *MYOM1* (Koebis et al., 2011), *TNNT2* (Philips et al., 1998) or *TNNT3* (Lin et al., 2006; Vihola et al., 2010; Yamashita et al., 2012). However, a recent report showed that CELF1 has no major role in alternative splicing regulation in differentiating myoblasts (Peng et al., 2015). Absence of CELF1 did not correct mis-splicing events in a DM1 mouse model (5-313^+/−^) (Kim et al., 2014). Because CELF1 is also known as a key regulator of mRNA translation and decay in the cytoplasm (Iakova et al., 2004; Vlasova St Louis et al., 2013), we propose that the cytoplasmic functions of Bru-3/CELF1 might have an important, and so far underestimated, impact.

Indeed, as revealed by our transcriptomic studies, a set of transcripts encoding sarcomeric proteins displays a reduced level dependent on Bru-3. This could hardly result from mis-splicing as no alternative splicing events have been reported for some of them (*Mlc2*, *TpnC47D*). We also tested by CLIP-PCR whether CELF1 that is re-localized to the cytoplasm of C2C12 myotubes binds to sarcomeric mature mRNAs, and found that *Actn*, *Tpm2* and *Pdlim5* sort as its targets. Importantly, a report by Wang et al. (2015) confirms our findings, showing that in mouse muscles, CELF1 interacts directly with a large set of mRNAs encoding sarcomeric proteins. Consistent with our observations on *bru-3* overexpression in flies, several sarcomeric transcripts are also affected by overexpressing *CELF1* (Wang et al., 2015), suggesting a general role of CELF1/Bru-3 in regulating the stability/storage of mRNAs encoding sarcomeric components.

### Potential dual role of Bru-3 in the sarcoplasm

**Fig. 7.**
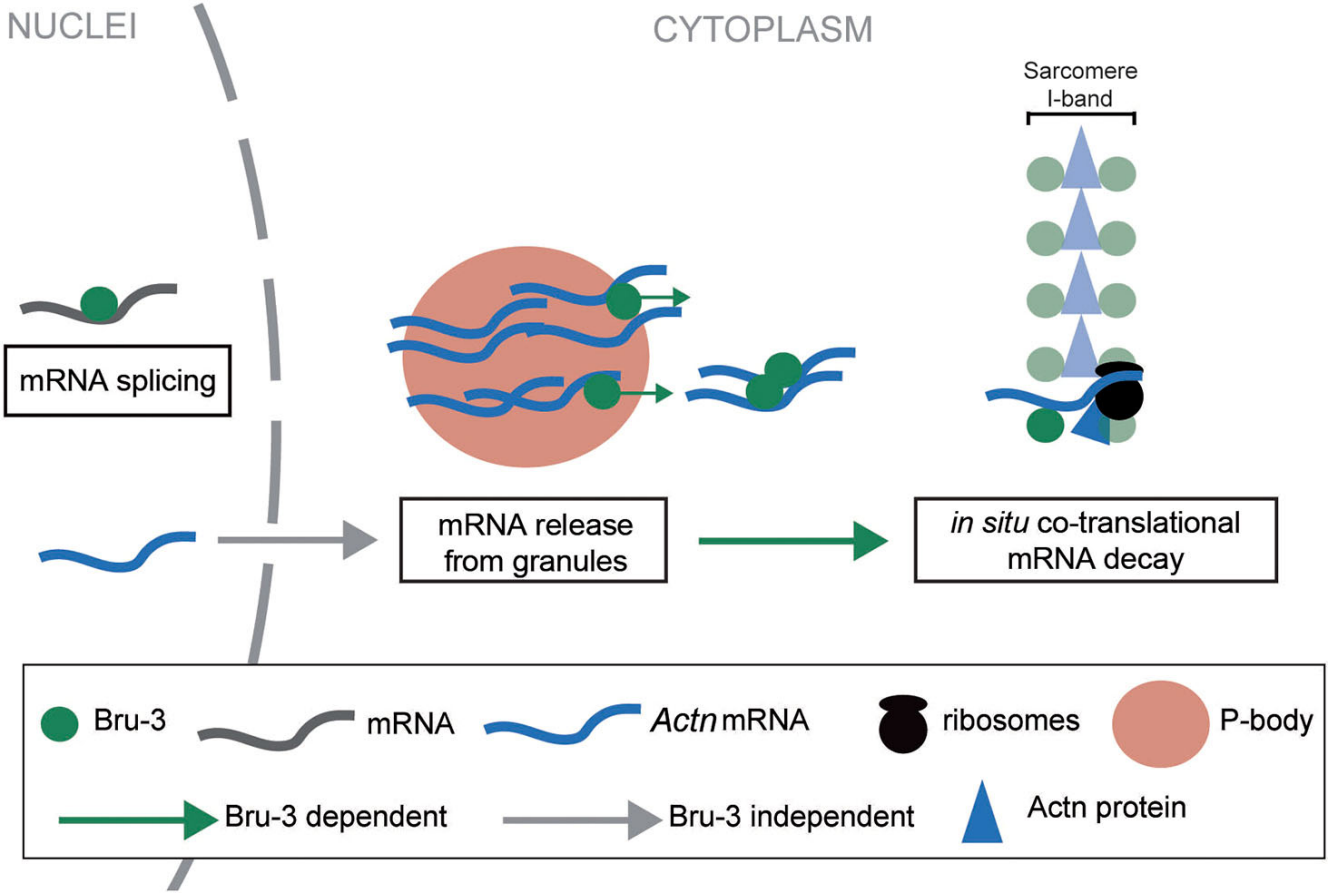
**Hypothetical model for dual** **Bru-3 role in the sarcoplasm.** Sarcomeric transcripts such as *Actn* transcripts are exported from the nuclei to the sarcoplasm independently of Bru-3. Before being translated, they are stored in cytoplasmic granules/P-bodies. Cytoplasmic Bru-3 associates with granules and promotes release of sarcomeric mRNAs, which then undergo *in situ* translation in sarcomeres. Bru-3 positively regulates this *in situ* translation and at the same time leads to co-translational mRNA decay. The newly synthesized proteins, such as Actn protein, are immediately incorporated to the sarcomeres. Thus, according to this model, the increased levels of cytoplasmic Bru-3 detected in Bru-3-overexpressing and DM1 contexts would have double impact on sarcomeric components. Bru-3 associated with granules would favor the release of sarcomeric transcripts from these storage sites. In parallel, Bru-3 associated with sarcomeres would promote *in situ* translation of released transcripts and their quick subsequent decay, leading to the reduction of sarcomeric RNA levels. In such a context, proper turnover of sarcomeric components will be affected, or at least inefficient, and could contribute to muscle weakening observed in DM1.

Bru-3 in the cytoplasm resides in granules detected around nuclei and also displays striated sarcomeric pattern. In the *bru-3* knockdown line, in which Bru-3 granules are almost absent, *Actn* mRNAs accumulate in muscle cytoplasm. Therefore, we hypothesize that Bru-3 could play a role in *Actn* mRNA release from granules. Also, the difference in ratios between transcripts and proteins of Actn in different contexts indicates a potential role for sarcomeric Bru-3 as a positive regulator of translation of mRNAs encoding sarcomeric proteins. One possibility is that Bru-3 regulates translation in muscle cells directly in sarcomeres, where it is localized. Taking all these observations into account, we propose a model for the dual cytoplasmic role of Bru-3 in muscles (Fig. 7).

According to this model, Bru-3 associated with stored mRNAs in granules/P-bodies around the nuclei would promote sarcomeric transcript release from these granules, whereas Bru-3 lying on both sides of the Z-line would positively regulate sarcomeric transcript translation and subsequent decay.

Several previously reported data support this hypothetical view. There is growing evidence that CELF1 colocalizes with P-bodies (Yu et al., 2013) and/or stress granules (Fujimura et al., 2008; Huichalaf et al., 2010; Vlasova-St Louis et al., 2013), and it has been reported that, in the absence of CELF1, *p21* (*CDKN1A*) mRNA (a CELF1 target) accumulates in stress granules concomitant to an increased stability and a decreased translational efficiency. Inversely, chemical disruption of stress granules increased *p21* translation while decreasing mRNA levels (Gareau et al., 2011). It has also been reported that Bru-3 could act as positive regulator of translation (Horb and Horb, 2010), that striated localization of Actn2 protein in cardiac myofibrils in the *Celf1* knockdown condition is affected (Blech-Hermoni et al., 2016), and that polyribosomes align along myosin thick filaments in differentiating myofibrils (Heywood et al., 1967; Allen and Terrence, 1968). Finally, our hypothesis fits well with a previously proposed translation/co-translational assembly model of Zarnescu and Gregorio (2013) and with co-translational RNA decay as described in yeast (Pelechano et al., 2015).

In conclusion, this study shows that the *Drosophila* CELF1 counterpart, Bru-3, contributes to DM1 muscle alterations, and could thus represent an interesting target for therapeutic strategies. Our data suggest a novel dual cytoplasmic role of Bru-3 in DM1 in the regulation of sarcomere component expression. Bru-3 associated with granules would favor the release of sarcomeric transcripts from these storage sites, while Bru-3 located within sarcomeres would promote *in situ* translation of released transcripts and their quick subsequent decay. As a consequence, transcript/protein ratios of sarcomeric components are affected and could impact on sarcomeric protein turnover, thus contributing to muscle structure defects and reduced muscle performance observed in DM1 patients. Further insights into Bru-3/CELF1 function in the sarcoplasm could shed more light on sarcomere maintenance and disturbed muscle function in DM1.

## MATERIALS AND METHODS

### *Drosophila* strains and crosses

All *D.* *melanogaster* stocks were grown and crossed on standard medium at 25°C. The site-specific inducible *UAS-960CTG* line that expresses 960-interrupted CTG has been described previously (Picchio et al., 2013). This line was recombined with the *Df(3L)Exel6119* line obtained from the Bloomington *Drosophila* Stock Center (BDSC, Bloomington, IN, USA) and is referred to as *UAS-960CTG,Df(bru-3)*. *bru-3^d09837^* [also cited as *UAS-bru-3(37)*], *bru-3^d09843^* [also cited as *UAS-bru-3(43)*] and *w^1118^;P{UAS-lacZ.B}melt^Bg4-1-2^* (cited as *UAS-lacZ*) were also obtained from the BDSC. *bru-3^KK111663^* (cited as the *UAS-bru-3RNAi* line) was obtained from the Vienna *Drosophila* RNAi Center (Vienna, Austria). Transgene expression was specifically driven in somatic muscles via the *Mef>mCD8GFP* line (a gift from A. Paululat, University of Osnabrück, Osnabrück, Germany) or the *Mef-Gal4* line (#27390, BDSC). Transgenic control lines were obtained by crossing transgenic lines with standard *w^1118^*. The driver control line was obtained by crossing the driver line with the *UAS-lacZ* line (*Mef>lacZ*).

### Motility test, muscle pattern assessment and measurements

The righting assay and muscle pattern assessment and measurements were performed as previously described (Picchio et al., 2013).

### Microarray analysis

Three independent total RNA isolations were performed on *Mef>bru-3(37)* and *Mef>lacZ* third-instar larvae using TRIzol reagent (Invitrogen) and the Agilent *Drosophila* gene expression microarray (G2519F, Strasbourg, France). Treeview (version 1.60, University of California at Berkeley) was used to confirm similarity (>70% Pearson correlation) between triplicates. The cutoff was set to a *P*-value <0.001 and a twofold increase/decrease. Differentially expressed genes were then classified according to the biological process they are involved in (Flybase). Microarray data are available in the ArrayExpress database under accession numbers E-MTAB-3231 and E-MTAB-1469.

### RT-qPCR

Total RNA was extracted from ∼100 µg of whole third-instar larvae using TRIzol reagent (Invitrogen). Then, 5 µg total RNA was treated with DNase I and reverse transcribed on a SuperScriptIII First Strand Synthesis System according to the provider's protocol (Invitrogen). Quantitative PCR was performed in duplicate in a final volume of 20 µl using Power SYBR Green PCR Master Mix (Roche, Applied Science) on a LightCycler 480 Real-Time PCR System (Roche, Applied Science). The primer combinations used are listed in Table S4. The relative quantifications of transcripts were obtained with the ΔΔCt method. Owing to the small number of samples (*n*=4-8), we opted for the nonparametric Kruskal–Wallis test to compare each genotype in order to determine whether their differences of distribution were significant. When appropriate, nonparametric Mann–Whitney tests were performed to compare control samples and samples of interest.

### *In situ* hybridization and immunofluorescent staining of *Drosophila* larval muscles

Third-instar larvae dissections were performed in 0.9% NaCl buffer containing 25 mM EDTA (except in the fiber contraction assay when EDTA was not added). Larval muscles were then fixed for 10 min in 4% formaldehyde on a plate, transferred to an Eppendorf tube, rinsed three times for 5 min in 1× PBS, 0.5% Tween (PBT) and blocked for 20 min in 1× PBT, 20% horse serum at room temperature. Incubation with primary antibody was performed for 2 h at room temperature with mouse monoclonal anti-Lamin C 28.26 (1:600; Developmental Studies Hybridoma Bank, Iowa, USA), rabbit anti-Bru-3 (1:1000; Millegen, Toulouse, France), rat monoclonal anti-Actn (1:200; BT-GB-276P, Babraham Technologies, Cambridge, UK). Muscles were then washed three times for 10 min with 1× PBT and incubated with fluorescent secondary antibodies (1:300; Jackson ImmunoResearch) for 1 h at room temperature and/or with phalloidin-TRITC (1:1000; P1951, Sigma-Aldrich) or Alexa Fluor 647-conjugated phalloidin (A22287, Invitrogen).

For standard sarcomeric protein transcript detection, *in situ* hybridization on larval muscles was performed on a TSA amplification system (Perkin-Elmer) according to the manufacturer's protocol. Immunostaining was performed in parallel to Dig detection. Mlc-1 and Actn Gold collection clones RE07220 and LD37956 were used to generate Dig-labeled RNA probes. For single-molecule FISH, custom Stellaris FISH probes were designed against the *Actn* open reading frame by utilizing the Stellaris FISH Probe Designer (Biosearch Technologies, Petaluma, CA, USA) available online at www.biosearchtech.com/stellarisdesigner. Larval muscles were hybridized with the *Actn* Stellaris FISH Probe set labeled with TAMRA Dye (Biosearch Technologies), following the manufacturer's instructions available online at www.biosearchtech.com/stellarisprotocols. Briefly, dissected muscles were fixed for 20 min in 4% paraformaldehyde and then rinsed twice for 5 min in 1× PBT. Tissues were then permeabilized with 75% ethanol for 5 min, then washed for 5 min in Stallaris Wash Buffer A (Biosearch Technologies). Hybridization was performed at 37°C in the dark for 4 h with 100 µl Stellaris hybridization buffer, containing 1 µl of 12.5 µM Stellaris probes. After hybridization, samples were rinsed in the dark for 30 min with Stellaris Wash Buffer A at 37°C and then for 5 min with Stellaris Wash Buffer B (Biosearch Technologies) at room temperature.

Dissected muscles were mounted in Vectashield with 4′,6-diamidino-2-phenylindole (DAPI). Images were acquired on a Leica SP8 confocal microscope. For quantification of Bru-3 in the different contexts, immunostainings were performed using the same dilution of antibodies. Images were acquired using the same settings on the Leica SP8 microscope throughout. Quantification of the signal was performed using Fiji software (https://fiji.sc/) following similar treatment of the picture.

### Western blot

Total protein was extracted from 0.1 mg of third-instar larvae using Buffer C (0.2 mM EDTA, 20 mM HEPES, 420 mM NaCl, 1.5 mM MgCl_2_, 25% glycerol, 0.2% Tween) supplemented with cocktail protease inhibitor (Complete Tablets Mini EDTA-free EASY Pack, Roche), with three to four samples/condition. Supernatant was collected after centrifugation (10 min, 18,300 ***g***, 4°C) and quantified using the Bradford assay. Samples were denatured in a loading buffer (β-mercaptoethanol) at 95°C for 10 min and resolved on 4-15% SDS-polyacrylamide gel (Mini PROTEAN TGX Gel, Bio-Rad, USA). To prevent formation of disulfide bonds and dissociate protein aggregates we applied iodoacetamide (IAA) treatment according to the manufacturer’s protocol (FOCUS Protein Alkylation kit, G-Biosciences, USA), with 40 μg of protein deposited per well. Separated proteins were electrotransferred onto nitrocellulose membrane using the Trans-Blot Turbo device (RTA Transfer Kit, Bio-Rad). Nonspecific sites were blocked for 30 min with Tris-buffered saline (TBS) containing 0.1% Tween 20 and 5% nonfat dry milk (blocking solution). The membranes were then incubated for 2 h at room temperature with rat monoclonal anti-Actn (1:10,000, BT-GB-276P, Babraham Technologies), and mouse monoclonal anti-alpha tubulin clone DM1A (1:10,000, T9026, Sigma-Aldrich) antibodies diluted in blocking solution. The membranes were washed in TBS-Tween 0.1% (three times for 5 min each and once for 15 min) and then incubated with peroxidase-conjugated secondary anti-IgG antibodies (Abcam) diluted 1/5000 in blocking solution. After rewashing in TBS-Tween 0.1% (three times for 5 min each), membrane peroxidase activity was tested by enhanced chemiluminescence (Pierce ECL2, Thermo Fisher Scientific) on a lumino-imaging analyzer (Chemidoc MP System, Life Science Research, Bio-Rad, France).

### Differentiation and immunofluorescent staining of C2C12 cells

C2C12 mouse myoblast cells were maintained in Dulbecco's modified Eagle's medium (DMEM) supplemented with 10% fetal bovine serum at 37°C in 5% CO_2_. For immunostaining, cells were grown on standard untreated coverslips. Myogenic differentiation was induced on confluent cells by switching culture medium to DMEM supplemented with 2% horse serum and insulin (5 µg/ml). Cells were held in differentiation medium for 10 days, with the medium replaced every 2 days. Differentiated or undifferentiated cells were rinsed in ice-cold PBS containing 1 mM MgCl_2_ and 1 mM CaCl_2_, then fixed for 30 min in ice-cold PBS containing 1 mM MgCl_2_, 1 mM CaCl_2_ and 4% paraformaldehyde. Cells were rinsed in PBS, permeabilized with 0.2% Triton X-100 in PBS for 15 min, re-rinsed with PBS, and blocked in PBS containing 3% bovine serum albumin (BSA). The blocked cells were incubated overnight at 4°C with primary antibodies (monoclonal anti-CELF1, clone 3B1, Santa Cruz Biotechnology) at 1 µg/ml in PBS containing 0.1% Tween and 1% BSA. Cells were rinsed three times in PBS containing 0.1% Tween and incubated for 2 h at 4°C with secondary antibodies (anti-mouse IgG coupled to Alexa Fluor 546, Invitrogen) at 1:2000 in PBS containing 0.1% Tween and 1% BSA. Cells were washed three times in PBS containing 0.1% Tween and counterstained with DAPI (0.01 µg/ml in PBS). Coverslips were mounted with ProLong Gold (Invitrogen) and observed using an Axio Imager M2 microscope (Zeiss) equipped with a 40× Plan-APOCHROMAT objective lens (Zeiss). Images were acquired on a CoolSNAP HQ2 CCD camera (Photometrics) and processed using ImageJ software (https://imagej.nih.gov/ij/).

### RNA CLIP

C2C12 were grown to confluence on 15 cm dishes and differentiated for 1 week in DMEM supplemented with 2% horse serum and insulin (5 µg/ml). For UV cross-linking, cells were rinsed in PBS and irradiated three times with 4000 µJ/cm² at 254 nm in a Stratalinker (Stratagene). Extracts were prepared by scraping in 300 µl PXL [1× PXL in PBS (no Ca^2+^ no Mg^2+^), 0.1% SDS, 0.5% nonidet P-40 (NP40), 0.5% sodium deoxycholate], then stored at −80°C. Beads used for immunoprecipitation were prepared by incubating 16 µg of antibodies (anti-CELF1, clone 3B1, Santa Cruz Biotechnology) or 16 µg of control IgG (I5381, Sigma-Aldrich) with 150 µl of a protein G-coupled paramagnetic bead suspension (Dynabeads Protein G, Novex) for 15 h at 4°C in PBST (PBS containing 0.01% Triton). Beads were blocked for 1 h in PBST containing 0.1% BSA and rinsed three times in PXL before use. For immunoprecipitation, cell extracts (300 µl) were mixed with 300 µl of PXL containing protease inhibitors (1/500, P8340, Sigma-Aldrich), DTT (2 mM), RNAsin (Promega, 1200 units/ml) and TurboDNase (Ambion, 40 units/ml). Genomic DNA was digested by incubating the samples for 10 min at 37°C with 1000 rpm agitation. Samples were centrifuged at 14,000 ***g*** for 15 min at 4°C. Supernatant (600 µl) was recovered and 20 µl was kept on ice (input fraction). Supernatants were added to 25 µl of either control IgG beads or anti-CELF1 beads. Immunoprecipitations were carried out at 4°C for 2 h. Beads were washed six times with stRIPA (50 mM Tris-HCl, pH 7.4, 1 M NaCl, 0.5% NP40, 1% sodium deoxycholate, 0.5 mM EDTA, 0.1% SDS, 2 M urea). Input fractions and washed beads were mixed with 47.5 µl of elution mixture [60 units/ml proteinase K (Thermo Fisher Scientific) and 200 µg/ml transfer RNA in stRIPA] and incubated for 30 min at 37°C. RNA was extracted with TriReagent (Sigma-Aldrich) and precipitated in propanol-2. RNA pellets were resuspended in 20 µl water, and 10 µl was reverse transcribed with random primers and Superscript II reverse transcriptase (Invitrogen). Complementary DNA (0.15 µl per reaction) was analyzed by RT-qPCR with SYBR Green PCR Mastermix (Applied Biosystems, 5 µl per reaction) and primer pairs at 0.5 µM in a total volume of 10 µl. The primer pairs used are listed in Table S5. Transcript enrichments in immunoprecipitated complexes were assessed by normalizing the Ct values measured in beads to those measured in input samples [Ct(Input)–Ct(Beads)]. Measures were taken from three independent cell dishes, and the enrichments obtained on specific (3B1 antibody-coupled) beads were compared with those obtained on control (IgG-coupled) beads, using a Student's *t*-test.

### *In silico* positioning of destabilizing motifs in 3′UTR sequences

3′UTR sequences were collected from UCSC (http://genome.ucsc.edu/) databases and BLASTed with either destabilizing motifs (Lee et al., 2010) (Table S3) or random motifs (Table S4) using a script purpose-developed in C++. Enrichments in the group of transcripts bound by CELF1 versus unbound transcripts group were tested for significance using Khi2 test without Yates' correction. BLAST positions for each sequence were sorted to generate hand-made diagrams in Excel.

### Statistics

All statistical analyses were performed using GraphPad Prism (version 5.02) software. Normality of the samples was assessed with a Kolmogorov–Smirnov test. One-way ANOVA (Newman-Keuls multiple comparison test or Dunn's multiple comparison post-test) were used for statistical comparisons of each pathologic line against its respective driver control line and/or transgenic control line. The smallest significance of both comparisons is reported on the graph. A Mann–Whitney test was only performed to compare the *DM1_960_* line with the *DM1_960_,Df(bru-3)* in rescue experiments, or the two *bru-3* overexpressing lines where reported. Results are reported as means±s.e.m., with *P*<0.05 considered statistically significant.

## Supplementary Material

Supplementary information
