# Supplementary material for: Bruno-3 regulates sarcomere component expression and contributes to muscle phenotypes of myotonic dystrophy type 1
Source: Dis Model Mech. 2018 May 21;11(5):dmm031849. doi: 10.1242/dmm.031849 (PMC5992612; doi:10.1242/dmm.031849)
Supplement: Supplementary information [file dmm-11-031849-s1.pdf]

SUPPLEMENTAL TABLES

Table S1 – Genes deleted in *Df(bru-3)* deletion in addition to *bru-3*

Click here to Download Table S1

Table S2 – Up and down-regulated genes by *bru-3* overexpression

Click here to Download Table S2

Table S3 – Random and destabilizing hexamers enrichment in 3'-UTR sequences of CLIP positive transcripts vs all genomic transcripts

Click here to Download Table S3

Table S4 – primers used for qPCR on larval samples

Click here to Download Table S4

Table S5 – Primers used for qPCR on C2C12 myotubes

Click here to Download Table S5

SUPPLEMENTAL FIGURES

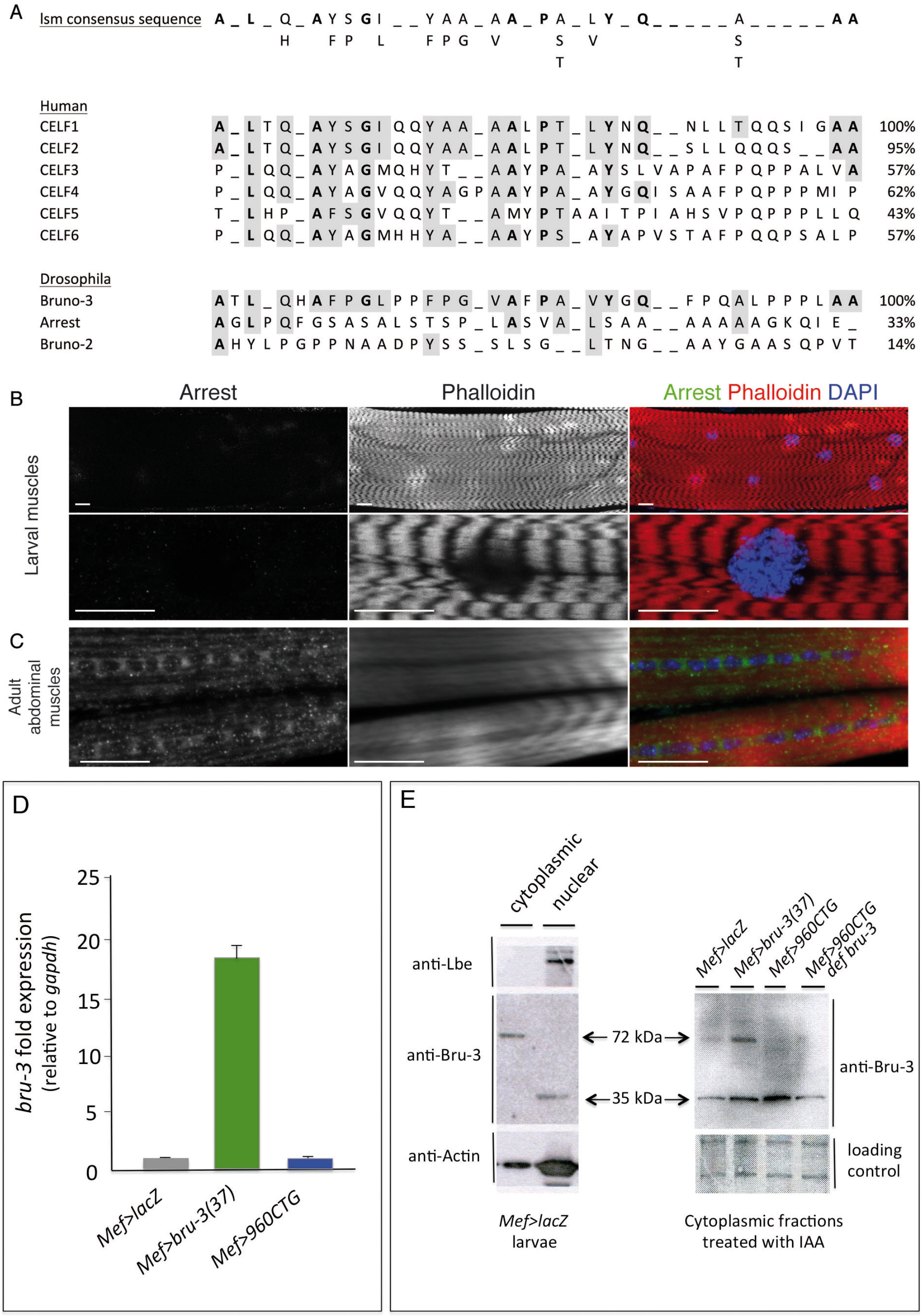

Disease Models & Mechanisms • Supplementary information

Figure S1. Evidences supporting *bru-3* as *Celf1* orthologue in larval muscles. (A) Sequence alignment of human CELFs lsm motifs with Brunos lsm motifs. Lsm consensus sequence was extracted from Delaunay et al., 2004. (B) Immunostaining of Arrest in larval muscles. Scale-bars represent 25 µm. (C) Immunostaining of Arrest in adult abdominal muscles. Scale-bars represent 10 µm. On these pictures, the rabbit anti-Arrest antibody was used (1:250, F. Schnorrer). (D) qRT-PCR analysis of *bru-3* transcript levels in control (*Mef>lacZ*), *Mef>bru-3(37)* and *Mef>960CTG* conditions. (E) Western blot analysis of Bru-3 protein expression in nuclear and cytoplasmic fractions isolated from muscle-enriched dissected *Mef>lacZ* 3rd instar larvae (left WB) and in IAA-treated cytoplasmic fractions isolated from *Mef>lacZ, Mef>bru-3(37), Mef>960CTG* and *Mef>960CTG; Df(bru-3)*. Anti-Lbe antibody detecting nuclear only protein Lbe is used as quality control for nuclear *versus* cytoplasmic fractions. Anti-Actin is used as loading control in the left WB panel. Note that about 10 times more of protein extract was loaded to detect nuclear Bru-3 protein (compare intensity of Actin bands). Because Bru-3 overexpression could potentially impact on muscle protein levels including sarcomeric Actin we opted to use Coomassie blue staining as loading control when using different genetic contexts influencing Bru-3 expression (right WB panel).

Disease Models & Mechanisms • Supplementary information

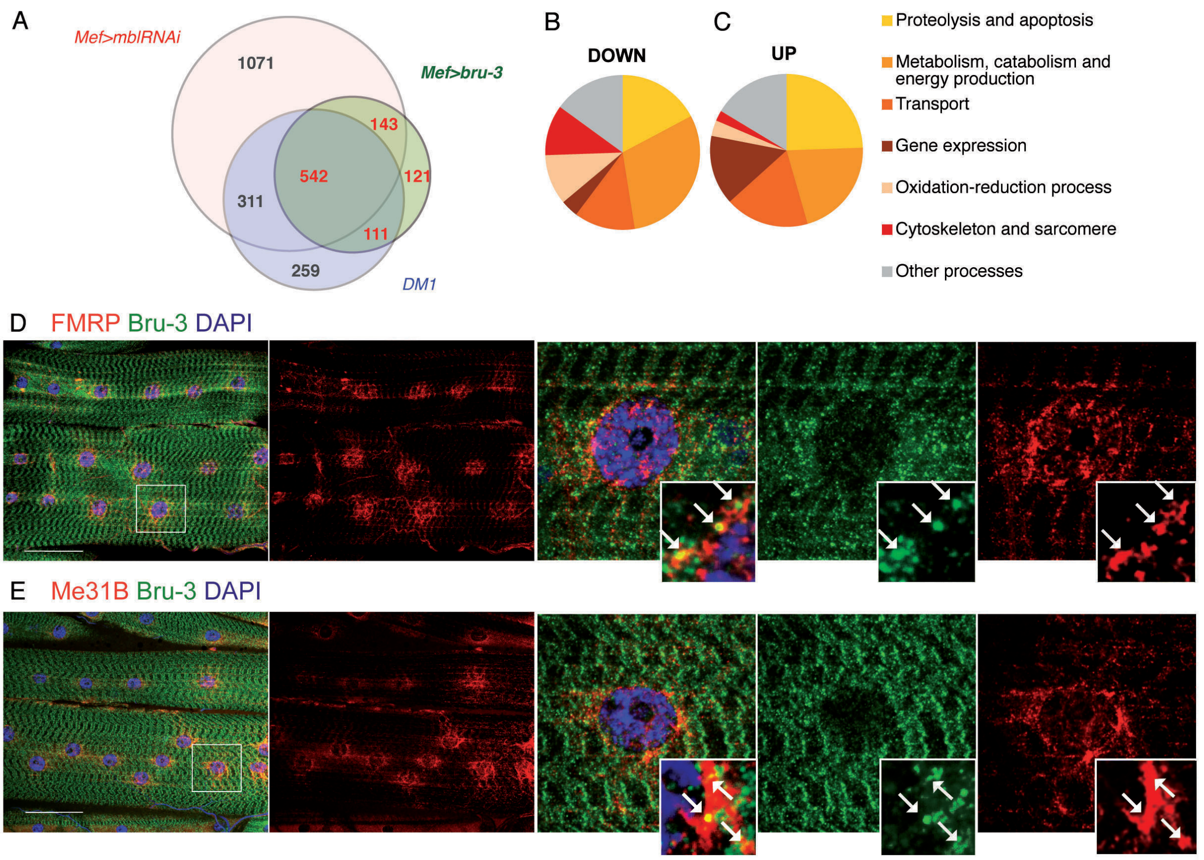

Figure S2. Global gene expression analysis of *bru-3* overexpression suggests a cytoplasmic function of Bru-3 in regulating sarcomeric transcript accumulation in P-bodies. (A) Venn diagrams of genes upregulated in *Mef>bru-3(37)* condition *vs. Mef>mblRNAi* and DM1 (*Mef>960CTG∩Mef>600CTG*) lines shows that about 90% of transcriptomic alterations caused by Bru-3 overexpression are common to DM1 and/or *mbl*-attenuated lines. The diagram was generated from a list of transcripts that are >1.5-fold enriched or depleted relative to the *Mef>lacZ* reference. (B-C) Pie chart recapping the Gene Ontology-based biological process distribution of downregulated (B) and upregulated (C) genes on the *Mef>bru-3(37)* microarray. Genes of unknown biological process and molecular function were not taken into account in these charts (n_{down} = 101; n_{up}=99). (D-E) P-body markers FMRP (D) and Me31B (E) are expressed around the DAPI-stained nuclei (blue) in muscle fibers and partially co-localize with Bru-3 (arrows). Scale bar 60 μm.

Disease Models & Mechanisms • Supplementary information

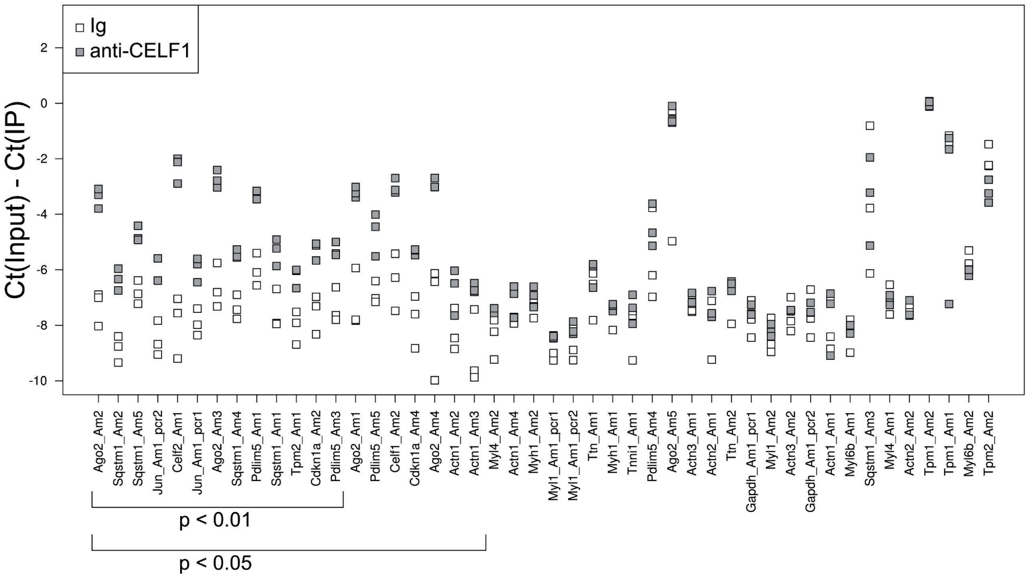

Figure S3. CELF1 can bind sarcomeric transcripts in C2C12 myotubes Results of CLIP experiments for all primer pairs tested.

Disease Models & Mechanisms • Supplementary information
